# Supplementary material for: Risk factors of reattempt among suicide attempters in South Korea: A nationwide retrospective cohort study
Source: PLoS One. 2024 Apr 18;19(4):e0300054. doi: 10.1371/journal.pone.0300054 (PMC11025816; doi:10.1371/journal.pone.0300054)
Supplement: S1 Table — (DOCX) [file pone.0300054.s002.docx]

**S1 Table. Suicide-Related Diagnoses and Categorization**

| **Initial inclusion** | **Exclusion** | **Finally selected code** | **Injury Category** |
| --- | --- | --- | --- |
| W65-W70, W73-W74, T75.1, Y21 | - W65(Drowning and submersion while in bath-tub) - W66(Drowning and submersi, on following fall into bath-tub) - W67(Drowning and submersion while in swimming-pool) - W68(Drowning and submersion following fall into swimming-pool) | W69, W70, W73, W74, T75.1, Y21 | Drowning |
| W13-W19, Y30-Y31 | - W14(Fall from tree) - W16(Diving or jumping into water causing injury other than drowning or submersion) - W17(Other fall from one level to another) - W18(Other fall on same level) | W13, W15, W19, Y30, Y31 | Fall down |
| W75-W76, R09.0,T71, Y20 | - W75(Accidental suffocation and strangulation in bed) | W76, R09.0, T71, Y20 | Hanging |
| Z91.5 |  | Z91.5 | Personal history of suicide attempt |
| S51, S60, S61, S66, S69 | with NN100 code | S51, S60, S61, S66, S69 | Wrist cutting |
| X60-X84 |  | X60-X84 | Intentional self-harm |
| Y28-Y29 |  | Y28-Y29 | Contact with blunt or sharp object, undetermined intent |
| T14.9,Y22-Y27, Y32-Y34 | with NN100 code | T14.9, Y22-Y27, Y32-Y34 | Injury, unspecified |
| F10-F19, T36-T60, T65, Y10-Y19 | - F10(Mental and behavioural disorders due to use of alcohol) - F12(Mental and behavioural disorders due to use of cannabinoids) - F14(Mental and behavioural disorders due to use of cocaine) - F15(Mental and behavioural disorders due to use of other stimulatnts, including caffeine) - F16(Mental and behavioural disorders due to use of hallucinogens**)** - F17(Mental and behavioural disorders due to use of tobacco) - T51(Toxic effect of alcohol) - Y15(Poisoning by and exposure to alcohol, undetermined intent) | F11.0, F13.0, F13.1, F18.0, F18.1, F19.0, F19.1, T42, T43  (with NN100 code) T36-T41, T44- T50, T52-T60, T65, Y10-Y14, Y16-Y19 | Drug intoxication |
| Z64.2, Z64.3 |  | Z64.2, Z64.3 | Problems related to seeking and accepting physical/behavioral, nutritional, and chemical/psychological interventions known to be hazardous and harmful |
| Y87 | - Y87.1(Sequelae of assault) | Y87.0, Y87.2 | Sequelae of intentional self- harm, assault and events of undetermined intent |
| R45.8 |  | R45.8 | Other symptoms and signs involving emotional state |
