# Supplementary material for: Risk factors of reattempt among suicide attempters in South Korea: A nationwide retrospective cohort study
Source: PLoS One. 2024 Apr 18;19(4):e0300054. doi: 10.1371/journal.pone.0300054 (PMC11025816; doi:10.1371/journal.pone.0300054)
Supplement: S2 Table — (DOCX) [file pone.0300054.s003.docx]

**S2 Table. Emergency Care-related Codes**

| Insurance code | Descriptions |
| --- | --- |
| AC100 | Emergency Management Charge - Emergency medical centers |
| AC101 | Emergency Management Charge - National emergency medical centers, Regional emergency medical centers |
| AC103 | Emergency medical care - Specialized emergency medical centers, Local emergency medical centers |
| AC105 | Emergency Management Charge - Local emergency medical centers |
| AC200 | Emergency Management Charge - General hospitals or emergency medical institutions, excluding emergency medical centers |
| NN100 | Psychiatric emergency treatment |
| V1100 | Emergency Management Charge - National emergency medical centers |
| V1200 | Emergency Management Charge - Regional emergency medical centers (Grade B) |
| V1210 | Emergency Management Charge - Regional emergency medical centers (Grade A) |
| V1220 | Emergency Management Charge - Regional emergency medical centers (Grade C) |
| V1300 | Emergency Management Charge - Local emergency medical centers (Grade B) |
| V1310 | Emergency Management Charge - Local emergency medical centers (Grade A) |
| V1320 | Emergency Management Charge - Local emergency medical centers (Grade C) |
| V1400 | Emergency Management Charge - Local emergency medical centers |
| V1500 | Emergency Management Charge - Regional emergency medical centers - Regional trauma centers (Grade B) |
| V1510 | Emergency Management Charge - Regional emergency medical centers - Regional trauma centers (Grade A) |
| V1520 | Emergency Management Charge - Regional emergency medical centers - Regional trauma centers (Grade C) |
| V1800 | Emergency Management Charge - Local emergency medical centers - Regional trauma centers (Grade B) |
| V1810 | Emergency Management Charge - Local emergency medical centers - Regional trauma centers (Grade A) |
| V1820 | Emergency Management Charge - Local emergency medical centers - Regional trauma centers (Grade C) |
