# Supplementary material for: Risk factors of reattempt among suicide attempters in South Korea: A nationwide retrospective cohort study
Source: PLoS One. 2024 Apr 18;19(4):e0300054. doi: 10.1371/journal.pone.0300054 (PMC11025816; doi:10.1371/journal.pone.0300054)
Supplement: S3 Table — (DOCX) [file pone.0300054.s004.docx]

**S3 Table. Comparison of the cohort with 2014 NEDIS data**

|  | | | | |
| --- | --- | --- | --- | --- |
| **Category** | **HIRA data (Our study) N=20614** | | **2014 NEDIS data (N=24811)** | |
|  | **n** | **%*** | **n** | **%** |
| Drowning | 855 | 4.15 | 275 | 1.10 |
| Fall down | 937 | 4.55 | 602 | 2.40 |
| Hanging | 1777 | 8.62 | 2042 | 6.20 |
| Personal history of suicide attempt | 766 | 3.72 | NA | |
| Wrist cutting | 801 | 3.89 | 2858 | 23.6 |
| Intentional self-harm | 538 | 2.61 |  |  |
| Contact with blunt or sharp object, undetermined intent | 591 | 2.87 |  |  |
| Injury, unspecified | 967 | 4.69 |  |  |
| Drug intoxication | 12663 | 61.43 | 14706 | 59.30 |
| Problem related to seeking and accepting physical, nutritional and chemical interventions known to be hazardous and harmful, Problem related to seeking and accepting behavioural and psychological interventions known to be hazardous and harmful | 2 | 0.01 |  |  |
| Sequelae of intentional self-harm, assault and events of undetermined intent | 10 | 0.05 | NA | |
| Other symptoms and signs involving emotional state | 1704 | 8.27 | 1328 | 5.40 |
| * The sum of percentage was not 100 since there were some cases duplicated category. | | | | |
